# Pioneer Factors in Animals and Plants—Colonizing Chromatin for Gene Regulation

**DOI:** 10.3390/molecules23081914

**Published:** 2018-07-31

**Authors:** Xuelei Lai, Leonie Verhage, Veronique Hugouvieux, Chloe Zubieta

**Affiliations:** Laboratoire de Physiologie Cellulaire et Végétale, CNRS, Univ. Grenoble Alpes, CEA, INRA, BIG, 38000 Grenoble, France; Xuelei.LAI@cea.fr (X.L.); Leonie.VERHAGE@cea.fr (L.V.); veronique.hugouvieux@cea.fr (V.H.)

**Keywords:** transcription factor, pioneer activity, cell fate transition, chromatin accessibility

## Abstract

Unlike most transcription factors (TF), pioneer TFs have a specialized role in binding closed regions of chromatin and initiating the subsequent opening of these regions. Thus, pioneer TFs are key factors in gene regulation with critical roles in developmental transitions, including organ biogenesis, tissue development, and cellular differentiation. These developmental events involve some major reprogramming of gene expression patterns, specifically the opening and closing of distinct chromatin regions. Here, we discuss how pioneer TFs are identified using biochemical and genome-wide techniques. What is known about pioneer TFs from animals and plants is reviewed, with a focus on the strategies used by pioneer factors in different organisms. Finally, the different molecular mechanisms pioneer factors used are discussed, highlighting the roles that tertiary and quaternary structures play in nucleosome-compatible DNA-binding.

## 1. Introduction

**Figure 1:**
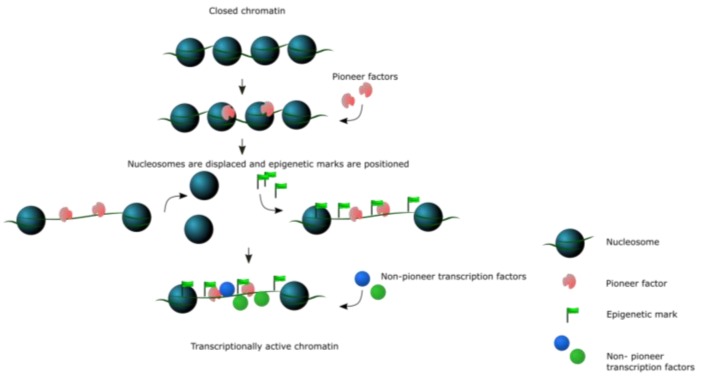
Activity of pioneer transcription factors. Pioneer factors bind nucleosomal DNA and open closed chromatin regions, e.g., by displacing nucleosomes, so that non-pioneer transcription factors can bind and regulate gene expression. In some cases, pioneer factors promote epigenetic marks deposition and render the ‘pioneered sites’ in an active state for a longer period of time (Table 1 and Table 2).

Transcription factors (TFs) are DNA-binding proteins that read genomic information to control gene expression in all organisms [1,2,3,4,5]. They achieve this by binding to their cognate DNA motif in gene regulatory regions, leading to either transcriptional activation or repression depending on whether transcription machineries are recruited or excluded. In prokaryotes, TFs recognize their DNA motifs with high specificity and affinity, suggesting that DNA sequence is the determining factor in TF function and gene regulation [6]. In contrast, TFs in higher eukaryotes often interact with other TFs in a combinatorial manner to ensure specificity and affinity [7]. By further recruiting ternary factors, such as epigenetic factors or other transcriptional machineries, eukaryotic TFs are able to establish robust temporal and spatial gene expression in response to environmental or cellular conditions and at different developmental stages. Compared to prokaryotic TFs, eukaryotic TFs confront another hurdle—namely, the complex structure of chromatin in which genomic DNA is wrapped around histone proteins to form nucleosomes, which is then further compacted to form higher-order structures [8]. Histone proteins can compete with TFs for DNA-binding, therefore limiting access to transcription factor binding sites (TFBS) [9]. This chromatin barrier therefore poses a significant challenge to the establishment of new gene regulatory networks, which is required, for example, during developmental phase transitions or organ specification. To overcome such obstacles, eukaryotes have evolved a unique set of TFs that are able to bind to their cognate motifs even when nucleosomes are present, subsequently priming the region for access by other DNA-interacting or modifying proteins. Collectively, these TFs are called pioneer factors (Figure 1).

The pioneer factor concept originated from in vivo footprinting studies, where researchers sought to determine which TFs were the first ones to bind a tissue-specific enhancer during embryonic development [10,11]. Two TFs that are important for endoderm development, FOXA1 and GATA4, were characterized as ‘pioneer factors’. Both have been shown to be able to engage silent heterochromatin, while endowing these regions with the competence for gene expression by allowing non-pioneer TFs to bind in the ‘pioneered sites’ [12]. Further in vitro biochemical studies have shown that recombinant FOXA1 and GATA4 are able to bind compacted chromatin and to open the local nucleosome-rich domains, even in the absence of ATP-dependent chromatin remodeling enzymes [13]. Several additional pioneer factors from different organisms have been identified in the last two decades (Table 1 and Table 2). In this review, we address how pioneer TFs are identified experimentally, through the common and distinct features of pioneer TFs from animals and plants and the strategies by which pioneer TFs bind and open chromatin. Additional reviews of pioneer TFs, focusing on different aspects of their activity, can be found elsewhere for further information on these key players in gene regulation [14,15,16,17,18,19,20,21,22].

## 2. Identification of Pioneer Factors—Biochemical and Genome-Wide Studies

### 2.1. Electrophoretic Mobility Shift Assays

Pioneer factors were originally identified as master regulators of cell fate and their ability to reprogram cell fate has been investigated at the molecular level. The extensive reprogramming of gene regulatory networks triggered by pioneer factors requires the opening and/or closing of different chromatin regions and the binding of nucleosomal DNA (Figure 1). Indeed, the ability to target a TFBS within a nucleosome has been a defining characteristic for pioneer factors. The canonical examples are FOXA1 and GATA4 pioneer factors, which were shown to be capable of binding to in vitro reconstituted nucleosomes that contain their TFBSs by electrophoretic mobility shift assays (EMSA). In these experiments, various liver-specific TFs were tested for their ability to bind to their TFBS on nucleosomes. Remarkably, it was found that only purified FOXA1 and, to a lesser extent, GATA4, but not other TFs, could bind to nucleosomal DNA. Further characterization showed that FOXA1 and GATA4 could open a local domain of compacted chromatin without ATP or ATP-dependent chromatin remodelers [13]. Since its successful application on FOXA1 and GATA4, EMSAs using reconstituted nucleosomes have been used to identify many pioneer factors [13,23,24,25]. These studies provide direct in vitro evidence of nucleosomal DNA binding, and have been used to characterize OCT4, SOX2, KLF4, and c-MYC, as well as FOXA1 and GATA4. In addition to in vitro band shift assays, genome-wide experiments have also been used to identify putative pioneer function.

For in vivo evidence of pioneer activity, genome-wide binding experiments, and correlation with chromatin states at the TFBS have been performed for different pioneer factors (Table 1 and Table 2). Due to technical advances in next-generation sequencing (NGS) techniques, many high-throughput approaches are now available to facilitate pioneer factor identification. Here, we summarize these NGS techniques such as chromatin immunoprecipitation followed by sequencing (ChIP-seq) and different variants of chromatin accessibility assays, which are used to correlate chromatin accessibility with TF binding.

### 2.2. ChIP-Seq and Variants

ChIP-seq has long been considered the technique of choice to map TFBSs in the genome for a given TF. Briefly, the protein of interest is cross-linked with the DNA, and the TF-DNA complex is purified through immunoprecipitation (IP) with an antibody. Subsequently, the protein-associated DNA is subjected to sequencing to identify the genomic regions bound by the TF. When compared with chromatin accessibility assay profiles (see below), it is possible to derive information with respect to whether or not a TF can target closed or poorly accessible chromatin, a key feature for pioneer factors. ChIP-seq has been successfully used for many TFs, and several ChIP-seq variant methods have been developed, which are capable of dealing with low-input materials and giving a high signal-to-noise ratio, among other advantages. Such variants includes ORGANIC (occupied regions of genomes from affinity-purified naturally isolated chromatin) [62], ChEC-seq (chromatin endogenous cleavage followed by sequencing) [63], CUT&RUN (cleavage under targets and release using nuclease) [64], SLIM-seq (short-fragment-enriched, low-input, indexed MNase ChIP) [65] and others.

### 2.3. Chromatin Accessibility Assays

The building blocks of chromatin are the nucleosome core particles, each consisting of approximately 147 base pairs of DNA wrapped around a histone octamer composed of four different core histones, generally, H3, H4, H2A, and H2B. Nucleosomes are arranged into ‘beads on a string’-like structures and are further compacted into highly condensed structures that confer transcriptional silencing [8,66,67,68,69,70]. Chromatin accessibility assays allow mapping of these open and closed regions of chromatin. As pioneer factors are the first TFs to engage in the closed chromatin, identifying chromatin-opening events by one of the various chromatin accessibility assays can give information on the potential pioneering capacity of the TF of interest.

Nuclease-based assays, such as DNase-seq [71,72,73] and MNase-seq, are the most widely used chromatin accessibility assays [74]. DNase preferentially cleaves nucleosome-depleted regions, whereas histone-bound DNA remains mostly uncleaved. The uncleaved DNA is precipitated out of solution, and the resulting ‘free’ DNA, which represents opened chromatin regions, can be sequenced and mapped to the genome. In contrast, MNase-seq takes advantage of both the endonuclease and exonuclease activity of MNase, which efficiently cleaves the ‘free’ DNA until it encounters obstacles such as nucleosomes—thus rendering the nucleosomal DNA protected and enriched. In this way, MNase-seq maps well-positioned nucleosomes in the closed chromatin regions. Both assays have been successfully used to identify pioneer factors. In particular, when combined with state-of-the-art computational algorithms, such as machine-learning techniques, DNase-seq allowed de novo identification of some 120 pioneer factors in mouse embryonic stem cells [33], including well-characterized pioneer factors, FOXA1, OCT4, and GATA TFs (Table 1), suggesting the robustness of such an approach. However, DNase-seq and MNase-seq have their intrinsic drawbacks. For example, both DNase and MNase display notable sequence-specific cleavage [75,76], meaning that potential bias still exists.

As alternative methods of determining chromatin accessibility, FAIRE-seq (Formaldehyde-Assisted Isolation of Regulatory Elements) and ATAC-seq (Assay for Transposase-Accessible Chromatin using sequencing) have been recently developed. FAIRE-seq has been used frequently due to its simplicity and lack of enzymatic digestion [77]. In brief, chromatin is crosslinked using formaldehyde in vivo, sheared by sonication, and extracted. The nucleosome-free DNA fragments are separated from the nucleosomal DNA and sequenced. In the last few years, ATAC-seq has grown in popularity and become the chromatin accessibility assay of choice due to its many advantages [78,79,80]. For example, ATAC-seq requires only a minimal amount of starting material, even being applicable for a single cell [81]. Briefly, nuclei samples are permeabilized by a low concentration of detergents that allow transposases, preloaded with NGS-sequencing compatible adaptors to enter the nuclei, cleaving the ‘free’ DNA and ligate adaptors. The resulting DNA is then amplified in a minimum number of PCR cycles and becomes readily suitable for NGS sequencing. The combination of techniques that both assess TF binding events and chromatin accessibility, before and after TF interactions, is key to determining whether or not a TF has pioneer activity in vivo.

### 2.4. Chromosome Conformation Capture Assays

In higher eukaryotes, the genome is organized into hierarchical folding of chromosomes in highly ordered three-dimensional structures. As revealed by chromosome conformation capture (3C) assays, chromosomes are divided into territories of A (active) and B (inactive) compartments [82,83,84,85], which are further partitioned into topologically associating domains (TADs). Within each TAD territory, gene interactions are enriched and largely stabilized by the architectural proteins, such as CTCF [86,87,88] and cohesion [89,90]. It has been well-established that genome organization contributes to gene regulation by fine-tuning chromatin looping between distal and proximal regulatory elements relative to transcriptional starting sites. For example, A/B compartments often preferentially interact with other compartments of the same type. Likewise, long-range gene interactions mainly occur within TADs and are insulated between TADs. This forms one of the major gene silencing mechanisms in higher eukaryotes. Therefore, a major gene activation event, such as transcriptional reprogramming during cell fate specification, is usually accompanied or preceded by A-B compartment switching, the breaking down of TAD boundaries, or establishment of communications between otherwise insulated TADs [91]. Given the widespread function of pioneer factors in cell fate specification, they likely play a role in re-shaping genome architectures. Indeed, a recent study by Stadhouders et al. showed that pioneer factors could drive topological genome re-organization at multiple architectural levels while also enabling gene regulatory rewiring during cell reprogramming, from somatic cells to pluripotent stem cells [92]. In this process, pioneer factors NANOG and SOX2 (Table 1) play a major role in triggering substantial changes in A/B compartmentalization and in facilitating the breaking down of TAD boundaries, as revealed by Hi-C datasets [92]. This case study provides de facto evidence that pioneer factors are capable of triggering the rearrangement of genome organization, and such activity is essential for large-scale gene expression reprogramming. Although the underlying mechanisms still remain elusive, it is appealing to hypothesize that intrinsic characteristics, such as TF oligomerization or interactions with canonical architectural proteins, like CTCF and cohesion, could allow pioneer factors to modify genome topology. Thus, experimental mapping of the 3D architecture of chromatin at different time points is critical for assessing pioneer activity.

In the last few years, many chromatin-conformation capture methods have been developed, such as 3C, 4C, 5C, Hi-C [83] and others, each with unique features and advantages [93,94]. These techniques are widely used in detecting high-order genome architectures and can be used for identifying and characterizing pioneer factors. Briefly, in 3C experiments, cross-linking agents, such as formaldehyde, is first applied to purified nuclei in order to capture protein (complex)-DNA interactions. The fixed chromatin is then digested with a restriction enzyme, followed by re-ligation of the digested DNA fragments. The re-ligation is usually performed in a highly diluted solution. This allows DNA sequences that are far in linear distance but proximal in space to be ligated, while minimizing ligation of random sequences. The ligation products are then purified and subjected to NGS library preparation and sequenced by deep NGS sequencing. In general, only a small fraction of the resulting sequencing reads contains conjunction sequences that come from long-range gene interactions captured by re-ligation—thus, 3C experiments require much greater sequencing depth and more sophisticated computational analysis to construct bona fide conjunction sequences compared with other NGS practices. In particular, when accessing chromatin looping mediated by a given TF in the context of pioneer factor identification, ChIA-PET [95] or Hi-ChIP [96] could be used. Both methods combine chromatin ChIP with 3C, allowing chromatin-looping identification mediated by a TF of interest.

### 2.5. Loss/Gain-of-Function Experiments and Inducible Systems

Transgenic experiments provide another means of assessing pioneer activity of a given TF. In loss-of-function experiments, for example, removal of a pioneer factor should directly reduce the accessibility of its target regions, whereas in gain-of-function experiments, ectopic expression of the factor should increase the accessibility of these regions. In many pioneer factor studies, characterization of loss-of-function and gain-of-function lines is usually applied. For example, a recent study by Jacobs et al. suggested that GRAINY, a highly-conserved TF with essential roles in epithelial cell-fate specification and wound healing in animals, is a pioneer factor. The authors showed that deletion and ectopic expression of GRAINY causes loss and gain of DNA accessibility, respectively, suggesting pioneer activity [57]. Alternatively, inducible systems are also commonly used in pioneer factor identification.

Inducible systems, combined with time point experiments, are a robust way to investigate pioneer activity of a given factor, but are not high-throughput and require expertise to be properly performed. Among the most widely used inducible systems are estrogen receptor or glucocorticoid receptor-mediated systems, which have been applied for pioneer activity determination of, e.g., EBF1 [97], PU.1 [47], PAX7 [35], Activator Protein 1 (AP-1) [53], and LEAFY COTYLEDON1 (LEC1) [61] (Table 1 and Table 2). For example, through using inducible systems, recent studies have been able to demonstrate that pioneer factors can bind before any changes take place on the epigenome, and not vice versa. A study from Li et al. examined the events directed by EBF1 in multipotent progenitor cells [97]. Using a time-resolved, genome-wide approach and applying an induction system in developmentally arrested *ebf1* mutant cells, they manage to elucidate the hierarchy of events. They showed that DNA demethylation is in fact preceded by the formation of chromatin accessibility, which is again preceded by EBF1 occupancy. In another study, Mayran et al. used an inducible version of PAX7 and studied the dynamics of the chromatin in a similar, time-resolved manner [35]. Strikingly, the binding of PAX7 to pioneer sites was detectable within 30–60 min but became stronger over three days, during which time the chromatin slowly became more accessible. The slow opening of chromatin at pioneered sites might be a common feature for pioneer factor-induced chromatin opening. The mechanisms behind this delay might be attributed to a slow response of chromatin remodelers, or the requirement for binding of co-factors. However, the true molecular mechanisms are, as yet, poorly understood, and further research is necessary to gain insight into these events.

### 2.6. A Cautionary Note

As described above, genome-wide techniques are a powerful tool for the investigation of pioneer factors. However, it is important to carefully interpret the data. Assessing the ability of a certain factor to bind nucleosomal DNA can be achieved by combining ChIP-seq or its variants with chromatin accessibility assays. However, overlapping peaks of protein-DNA binding and closed chromatin do not indisputably prove that a factor can bind closed chromatin, as these interactions may be indirect. In addition, samples must be taken at the same developmental stage from the same tissue/cell population to avoid misinterpretation of TF binding and chromatin status. Not being able to detect a perfect overlap between chromatin accessibility and DNA binding does not necessarily indicate that a TF is not a pioneer factor, either. A recent study on GR-responsive enhancers showed that nucleosomal regions can sometimes show hypersensitivity to DNase I, in contrast to the idea that hypersensitivity reflects absence of nucleosomes [98]. A study on FOXA showed that previous attempts to detect nucleosomes by MNase digestion had failed to find differences in nucleosome occupancy in the absence or presence of FOXA1 and FOXA2, which was most likely due to the over-digestion by MNase [99]. Therefore, it is important that a combination of genome-wide and in vitro techniques, such as nucleosome reconstitution and EMSAs, is used to better verify pioneer activity.

## 3. Pioneer Factors in Animals and Plants

### 3.1. Mammalian Models

The idea of ‘pioneer factors’ was originally proposed based on functional studies that showed that the TFs FOXA1 and GATA4 were able to potentiate liver organogenesis from endoderm cells. This functional feature, namely, the ability to program or reprogram cell fate, was associated with the activation of previously silent target genes. In the last decade, many key TFs involved in cell fate specification have been identified as pioneer factors in mammals. These pioneer factors act as master regulators of major cellular events, including cell fate programming from embryonic cells to differentiated cell types, re-programming from somatic cells to pluripotent cells, and direct cell conversion or trans-differentiation, such as from fibroblasts to muscle cells (Table 1).

The differentiation of embryonic cells to distinct cell types in early embryonic development requires a dramatic reprogramming of gene expression patterns. Pioneer factors play a critical role in establishing competence for many different cell fate specification programs, for example, PAX7 in pituitary melanotrope development and PU.1 in myeloid and lymphoid development, among many others (Table 1). Cell types can also be reprogrammed into pluripotent stem cells (PSCs) that consequently have the ability to re-differentiate in all cell types, through the transfection of a handful of pioneer TFs. For example, OCT3/4, SOX2, KLF4, and c-MYC, collectively called the Yamanaka factors, were the first identified set of TFs with this ability. Together, they are sufficient to trigger the endogenous expression of downstream pluripotent factors, leading to re-programming of mouse- and human-derived fibroblasts into induced PSCs [100,101]. Among the Yamanaka TFs, OCT3/4, SOX2, and KLF4 are prominent examples of pioneer factors which have been extensively characterized due to their cell type reprogramming activity, whereas c-MYC does not seem to act as a true pioneer TF [22].

Pioneer TFs are also able to directly reprogram cells by switching somatic cells to a different type of somatic cell without passing through an intermediate pluripotent stage. For example, the combination of PU.1 and C/EBPα/β is sufficient to convert fibroblasts to macrophage-like cells [102], in which both TFs act as pioneer factors. The combination of GATA4, MEF2C, and TBX5 is able to trigger the induction of cardiomyocyte-like cells from fibroblasts [103,104], in which at least GATA4 acts as pioneer factor. Three TFs, ASCL1, BRN2, and MYTLl are examples of TFs capable of inducing trans-differentiation across germ layers. They are able to generate functional glutaminergic neurons from fibroblasts. In this process, ASCL1 acts as a pioneer factor and plays a central role in initiating trans-differentiation. ASCL1 alone is sufficient to induce immature glutaminergic neurons cells, but not BRN2 or MYTLl [105]. Other examples of trans-differentiation across germ layers are the induction of hepatocyte-like cells from fibroblasts by the ectopic expression of one of the FOXAs (FOXA1, FOXA2, or FOXA3) with HNF4 [106], or ectopic expression of FOXA3, GATA4, and HNF1a in combination with the inactivation of p19^Arf^, a tumor suppressor [107]. In these processes, the FOXA and GATA TFs are acting as pioneer factors. Thus, in mammals, pioneer activity has been identified in a number of high-level master regulators of cell fate, based on functional, genome-wide, and biochemical studies.

### 3.2. Plant Pioneer Factors

Our knowledge of pioneer activity in plant species is much more limited. A few TFs have been described as potential pioneer factors in plants, including LEC1, LEAFY (LFY), APETALA1 (AP1), and SEPALLATA3 (SEP3) (Table 2). LEC1 is a master regulator of embryo development and was shown to promote the initial establishment of active chromatin at the gene *FLOWERING LOCUS C* (*FLC*) [61]. *FLC* is a floral repressor which, during cold winters, is epigenetically repressed by Polycomb repressive complex 2 (PRC2) in a process called vernalization [108,109]. The resulting repressive epigenetic state allows plants to flower in spring but needs to be reset in the offspring. LEC1 is homologous to the B subunit of the heterotrimeric mammalian pioneer NF-Y TFs, which have been characterized as a pioneer factor in mammals [33,46,110,111]. NF-Y TFs can access their TFBSs in Polycomb-silenced domains [110]. This could promote chromatin accessibility and trigger active histone modification, as has been proposed for LEC1 [46,110,111].

LFY acts as a master regulator of flower development [112] and was shown to be able to access closed chromatin regions in a genome-wide scale analysis [59]. LFY has two domains—a Sterile Alpha Motif (SAM) oligomerization *N*-terminal domain, and a *C*-terminal novel helix-turn-helix DNA binding domain (DBD). The SAM domain does not affect DNA binding in vitro, but is required to fully complement the *lfy-12* mutant, suggesting that oligomerization is required for LFY function and that it potentially plays a role in pioneer function. ChIP-seq experiments performed on 2-week-old seedlings expressing either a LFY or LFY SAM-domain mutant impaired in oligomerization revealed a strong reduction of binding when the SAM domain was impaired. Comparison of ChIP-seq data with DNase-seq data performed in the same conditions suggested that LFY was able to bind to closed chromatin regions, and that the SAM domain was required for this binding [113]. In addition, LFY interacts with SPLAYED (SYD) and BRAHMA (BRM) [114], which are ATPase components of SWI2/SNF2 chromatin-remodeling complexes and are able to evict well-positioned nucleosomes. These two factors likely play an important role in LFY pioneer activity.

The MADS-box family TFs, AP1 and SEP3, have also been described as potential pioneer factors [60]. These TFs have protein–protein interaction/oligomerization domains, in addition to the core MADS-box DNA-binding domain. AP1 is an important regulator of floral meristem identity in Arabidopsis, and has additional roles as a homeotic regulator of sepal and petal identity [115]. SEP3 is a mediator of higher-order complex formation during floral organogenesis, and is thus a key regulator of floral organ identity [116,117,118]. Pioneer activity for AP1 and SEP3 was first suggested by time course experiments correlating chromatin states with AP1 and SEP3 binding. Genome-wide kinetic analysis, from the meristematic stages to the floral organ differentiation stages, was performed to assess the binding activity of AP1 and SEP3, as well as the concomitant chromatin status. These experiments showed that the binding of SEP3 and AP1 preceded the increase in DNA accessibility at their binding sites, suggesting AP1 and SEP3 pioneer activity. Similar to LEC1 and LFY, the ability to oligomerize seems to be important for the pioneer activity of AP1 and SEP3, as it has been shown in the case of SEP3 that reduction in its oligomerization efficiency correlated with a decrease in gene activation at certain nucleosome-rich loci [119]. Furthermore, physical interactions between SEP3, AP1, and chromatin remodeling factors have been demonstrated via immunoprecipitation mass spectrometry and yeast 2-hybrid experiments [114,120,121,122]. Overall, the function of plant pioneer TFs has parallels with their mammalian counterparts—namely, the role of these factors in cell differentiation and reprogramming during developmental transitions. As described below, the molecular mechanisms of animal and plant pioneer factors share common features, but also exhibit distinct characteristics in their mode of action.

## 4. Mechanism of Action

Pioneer TFs from animals and plants share common characteristics, namely, the ability to bind closed regions of chromatin and to trigger the opening of these regions, rendering them competent for the binding of other factors and/or gene expression. Different mechanisms have been proposed for pioneer factor-DNA binding in nucleosome-rich regions of chromatin. These include the ability to mimic linker or core histones, the capacity to bind in a nucleosome-compatible manner to a single face of the DNA, and the use of oligomerization to increase binding affinity to outcompete nucleosomes at their cognate binding sites. Upon successful binding, opening closed regions of chromatin may occur either through the direct displacement of nucleosomes or through the recruitment of chromatin-remodeling proteins and complexes.

**Figure 2:**
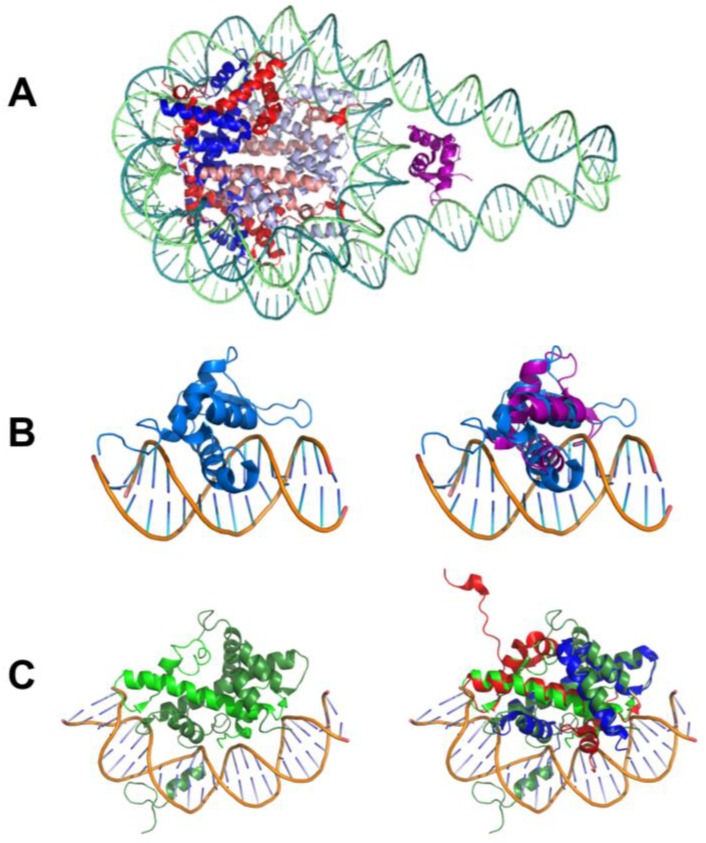
Structural comparison of histones and pioneer transcription factors (TF) showing histone mimicry. (**A**) Nucleosome structure including histone H1 (PDB 5NL0) shown as a cartoon with each histone colored uniquely—H1 in purple, H2A in red, H2B in blue, H3.2 in light blue and H4 in pink. H1 binds along the dyad axis, helping to stabilize the linker DNA. (**B**) Left, crystal structure of FOXO1 (PDB 3CO6) shown in blue bound to DNA. Right, overlay of FOXO1 (blue) and linker histone, H1 (purple). FOXO1 not only has the same fold as linker histone H1, but also binds DNA site specifically. (**C**) Left, structure of NF-Y transcription factor (PDB 4AWL) in complex with DNA. The protein subunits NF-YA, NF-YB and NF-YC are colored green and shown as a cartoon. Right, overlay of NF-Y (green) and histone H2A (red) and histone H2B (blue) dimer. NF-Y adopts the same fold as the H2A-H2B heterodimer while binding DNA site specifically.

A number of identified pioneer TFs possess histone-like folds, suggesting that histone mimicry may play an important role in pioneer function (Figure 2). For example, FOXA1, FOXE2, and FOXO [13,15] have a winged helix-turn-helix fold, structurally similar to the linker histone, H1, and have been shown to displace H1 [99] (Figure 2A,B). In addition to the highly conserved forkhead DBD, the FOXA subfamily has four transcription activation domains—two N-terminal and two C-terminal [123]. FOXA1 prefers binding to bent and nucleosomal DNA [124,125]. The C-terminal region of FOXA1 has been shown to interact with core histones, likely further facilitating protein binding in regions already occupied by histones and helping to anchor the TF even in inaccessible nucleosome-rich chromatin regions, and possibly aiding in their displacement [13,125,126]. Another example of histone mimicry is observed for the nuclear transcription factor Y (NF-Y), which are trimers made up of three subunits, NF-YA, NF-YB, and NF-YC. NF-Y from mammals and the seed-specific NF-YB TF, LEC1, exemplify this mechanism in which the TF adopts a fold structurally similar to core histones, H2A and H2B (Figure 2C). NF-YB and NF-YC form a heterodimer, similar to the histone fold domains of H2B and H2A, with NF-YB containing the sequence-specific DNA-binding domain [46]. NF-Y in mammals has been shown to promote chromatin accessibility [46,127,128,129,130].

### 4.1. Nucleosome-Compatible Binding and High Affinity

Another proposed mechanism distinct from histone-fold mimicry is single-side DNA binding that is seen in the mammalian pioneer structurally unrelated TFs, OCT4, SOX2, and KLF4 [30]. In plants, the putative pioneer factor, LFY, also exhibits a single-side DNA binding mode [131]. By preferentially binding to one side of DNA, these pioneer factors do not need to displace histones for binding their cognate sites. In addition, the bend of DNA wrapped around the histone octamer has been postulated to favor the binding of certain factors, such as SOX2 and OCT4 [30].

Oligomerization and cooperative binding, which increases DNA-binding affinity, has been hypothesized to play an important role in allowing pioneer factors to bind their cognate sites even in relatively inaccessible nucleosome-rich chromatin regions. For example, the mammalian pioneer factors OCT4, SOX2, and KLF4 are all able to oligomerize, increasing their binding affinity. A similar mechanism is likely used in plants. As mentioned previously, Arabidopsis LFY requires oligomerization activity in order to bind to low affinity LFY binding sites in closed chromatin regions [59]. Similarly, MADS-box TFs are able to tetramerize and bind DNA cooperatively, increasing their DNA-binding affinity [119,132]. In addition, a mechanism similar to histone octamer binding in which DNA wraps around the MADS-box tetramer has been proposed for plant MADS-box TFs, although this is highly speculative [133]. The combination of binding modes compatible with nucleosomal DNA or similar to histone octamer binding, coupled with high affinity due to cooperativity effects, may act as a general mechanism for pioneer activity, at least for a subset of pioneer factors. Taken together, the combination of histone mimicry or preferential binding to bent or nucleosomal DNA coupled to high-affinity sequence-specific DNA binding is likely required for the initial pioneer factor-chromatin interaction.

### 4.2. Pioneer Factors Recruit Chromatin Remodelers 

While histone mimicry can result in the direct displacement of histones, some pioneer factors open closed chromatin regions via the recruitment of chromatin remodelers. In mammals, one of the common chromatin remodelers that can be recruited by several pioneer factors, in most cases through direct physical interactions, is the ATPase BRG1 of the BAF complexes. For example, GATA1 has been shown to recruit BRG1 to closed chromatin regions and create accessible sites for secondary factors, such as TAL1, for downstream transcriptional activation, required for differentiation of human hematopoietic stem cells to erythrocytes [134]. Further examples include OCT4, which requires the BRG1 to shape chromatin accessibility and facilitate secondary TF binding during cellular reprogramming and early mouse development [31], and GATA3, which requires BRG1 to create accessible chromatin in the mesenchymal-to-epithelial transition [29]. Interestingly, the forkhead transcription factor FOXD3 has also been shown to recruit BRG1 for creating active enhancers during the differentiation of embryonic stem cells to epiblast cells in mice [135]. In plants, SWI2/SNF2 ATPases SYD and BRM seem to play an important role for the putative pioneer activity of LFY, AP1, and SEP3 [114]. In addition, AP1 and SEP3 have been shown to recruit additional chromatin remodelers, such as CHR11 and CHR17 [114,120]. Therefore, binding closed chromatin followed by recruitment of remodeling complexes is a plausible mechanism, enabling many pioneer factors to achieve histone displacement and subsequent chromatin opening, while also allowing the binding of other non-pioneer TFs.

### 4.3. Pioneer Factor Binding and Methylation of DNA and Histones

Besides binding to nucleosomal DNA, pioneer factors have been shown to be able to bind sites that are transcriptionally silenced by DNA methylation. DNA-methylated binding sites, and in particular, CpG methylated sites, are often regarded to be inaccessible to canonical transcription factors [136]. However, several pioneer factors, like PAX7, have been shown to be insensitive to CpG methylation and able to bind irrespective of DNA methylation status [35]. For p53, it has even been shown to exhibit enhanced binding to certain methylated sites [137]. Whether all pioneer factors exhibit a preference, or at least insensitivity, to the methylation of DNA is still to be determined.

### 4.4. Pioneer Factors Impact the Epigenetic Landscape

After the binding and opening of closed chromatin regions, some pioneer factors are able to facilitate the creation of long-term accessible chromatin regions to non-pioneer factors. To achieve this, pioneer factors promote the deposition of permissive histone modifications, such as H3K27ac3, and/or elimination of repressive histone markers, such as H3K27me3. For example, pioneer factor TCF-1 can erase H3K27me3 and H3K9me3 repressive marks near TCF-1 bound sites during T cell development in mice [36] (Table 1). In plants, LEC1 promotes active histone marker H3K36me3 deposition, and counteracts the effect of PRC2 by eliminating the repressive histone marker H3K27me3 [61]. Further examples can be found in Table 1 and Table 2. Compared with commonly found direct physical interaction between pioneer factor and chromatin remodelers, epigenetic editing facilitated by pioneer factors are likely to be an indirect and ancillary effect of pioneer activity. For example, the pioneer factor ESRRB promotes histone acetylation near its bound sites during pluripotent cell reprogramming, with such activity requiring LIF-dependent engagement of acetyltransferase p300 [58].

Up to now, no pioneer factor has been discovered to be able to have unrestricted access to all states of heterochromatin. In fact, pioneer factors exhibit cell specificity, being able to bind sites in certain cell types, but not in others [138]. For the Yamanaka factors, it was shown that the histone modification H3K9me3 is a barrier that restricts these pioneer factors from binding to heterochromatin [139]. Thus, the effects of histone modifications on pioneer TFs need to be explored in more depth, and it is likely that the interplay between histone-modifying enzymes and pioneer factors will reveal new complexities important for proper gene regulation.

## 5. Perspectives and Challenges

Pioneer factors are highly important in the development of eukaryotes. What strategy they use to overcome the difficulty of activating silenced genes in heterochromatin regions is a fundamental biological question. In the last few years, the knowledge of the mechanisms of action of these factors in human and animal systems has grown tremendously, facilitated by the rise of genome-wide techniques. It has become clear that different pioneer factors have different modes of action—how they interact with closed chromatin (e.g., only naïve or methylation independent), the way they allow opening of the chromatin (histone-mimicry or recruitment of chromatin remodelers), and how they help establish stable epigenetic marks.

While the majority of research on pioneer factors has been performed on mammalian systems, pioneer factors are starting to be identified in other eukaryotes. The idea of pioneer factors in plants only emerged several years ago. Interestingly, amongst the currently proposed plant pioneer factors, AP1, SEP3, LFY, and LEC1, only the latter shows homology to an animal pioneer factor. AP1 and SEP3 are MADS-box proteins, a protein family that does not appear to act as pioneer factors in animals, and LFY is an orphan protein that can only be found in plants. On the other hand, it appears that most of the animal pioneer factors belong to TF families that are absent from plants. For example, the forkhead box proteins that the different FOX pioneer factors belong to, and the Paired-Box TFs that include PAX7, are not found in plants.

Some mammalian pioneer factors belong to large families of TFs that are also present in plants, but they have not yet been identified as having a pioneer role. These factors include MYOD1 and ASCL1 of the bHLH TF family (more than 160 members in *Arabidopsis* [140]) and ZELDA of the C2H2 zinc finger family (more than 170 members in *Arabidopsis* [141]). The GATA TFs have 29 members in the *Arabidopsis* genome [142], and are involved in important plant developmental processes, such as germination, flowering, and senescence [142,143]. Meanwhile, animal GATA TFs appear to display pioneer activity by virtue of recruitment of the SWI/SNF chromatin remodeling ATPase BRG1 [29,134]. To our knowledge, no interactions between the plant GATAs, the plant SWI/SNF ATPases BRM and SYD, or other remodelers have been reported, leaving open the question of whether pioneer factors exist among the plant GATA TFs.

Many animal pioneer factors have a function in embryogenesis. When it comes to putative plant pioneer factors, it is important to realize that plants and animals differ greatly in this process. All organs are present after embryogenesis in animals, but organogenesis takes place throughout the plant life cycle. The putative pioneer factors in plants currently described are all involved in the developmental transition from vegetative to reproductive development. This switch requires fine-tuned remodeling of the chromatin architecture, as it is essential for the survival of the plant to not flower prematurely. Therefore, it is not unlikely that plants have evolved different pioneer factors to engage in important phase transitions during their lifetime. Hence, plant and animal pioneer factors might exhibit different modes of action in terms of both structure and function, and whether this is indeed the case will require further research.

## Figures and Tables

**Table 1:** Pioneer factors in animals [26].

Pioneer TFs	Species	Organ/Cell Type	Biological Process	Identification	Pioneer Activity	Co-Factors	Epigenetic Effects	Other Features and Cautious Notes	References
Forkhead box TFs	Human and mouse	Many, such as endoderm	Cell differentiation and organogenesis	EMSAs and ChIP-seq	Resembles H1 and binds nucleosomal DNA	N/A	N/A (not applicable)	PHA-4 recruits Pol-II [27]; FOXA2 recruits nucleosome disassembly complexes [28]	[13,27]
GATA family	Human and mouse	Many, such as endoderm	Cell differentiation and organogenesis	EMSAs	Binds nucleosomal DNA and create accessible chromatin	BRG1 of BAF complexes	N/A	N/A	[11,13,29]
OCT4	Human and mouse	Embryonic cells or fibroblast	Embryonic development or reprogram somatic cells to pluripotent cells	Activity of reprogramming cell fate; ATAC-seq, ChIP-seq and DNase-seq	Open closed chromatin by recruiting BRG1	BRG1	Facilitate H3K4me1/2/3, H3K9ac and H3K27ac deposition	N/A	[30,31]
SOX2	Human and mouse	Embryonic cells or fibroblast	Embryonic development or reprogram somatic cells to pluripotent cells	DNase-seq; Activity of reprogramming cell fate specification	Binds nucleosomal DNA and create accessible chromatin	N/A		N/A	[30]
KLF4	Human and mouse	Embryonic cells or fibroblast	Embryonic development or reprogram somatic cells to pluripotent cells	Activity of reprogramming cell fate; DNase-seq	Binds nucleosomal DNA and create accessible chromatin	N/A		N/A	[30,32]
NRF1	Human and mouse	Embryonic cells	Cellular growth	DNase-seq; ChIP-seq	Create DNase-hypersensitive sites upon binding	N/A	N/A	Sensitive to DNA-methylation [22]	[33,34]
PAX7	Mouse	Melanotrope	Specifies intermediate pituitary melanotrope cell identity	ATAC-seq	Open closed chromatin	p300	Reduce DNA methylation and acquire epigenetic memory	Binds enhancers rapidly, but gene activation are slower [35]	[35]
TCF-1	Human and mouse	T cells	T cell lineage establishment	ATAC-seq	Open closed chromatin	N/A	Erase H3K27me3 and H3K9me3	N/A	[36]
ASCL	Human and Mouse	Glioblastoma Stem Cells or embryonic stem cells	Neurogenesis, conversion of fibroblasts into induced neuronal cells [37]	ATAC-seq, MNase-seq and ChIP-seq	Open closed chromatin	N/A	Induce H3K37ac deposition	N/A	[37,38,39]
C/EBPα	Mouse	B cells	Pre-B-cell to macrophage trans-differentiation	MNase-seq and ChIP-seq	Create de novo chromatin accessibility	Cooperative binding with PU.1	N/A	N/A	[40]
EBF1	Mouse	B cells	Lymphopoiesis	DNase-seq	Promoted chromatin accessibility	N/A	Promote DNA demethylation	N/A	[41]
NeuroD1	Mouse	Neuron	Neuronal specification	ChIP-seq and FAIRE-seq	Conversion of heterochromatin to euchromatin	N/A	Promote H3K27ac and reduce H3K27me3	N/A	[42]
ER and GR	Human	Many	Many	DNase-seq and ChIP-seq	Enhance binding of pioneer factor FOXA1	SWI/SNF complex [43]	N/A	Pioneer activity under strong debate [18]	[44]
PR	Human	Breast cancer cells	Breast tumorigenesis	DNase-seq, MNase-seq and ChIP-seq	Initiate chromatin binding and remodeling	N/A	N/A	N/A	[45]
NF-Y	Mouse	Embryonic cells and neurons	Maintenance of embryonic cell identity	ChIP-seq	Mimic histone proteins	N/A	Promote H3K4me1 and H3K27ac deposition and reduce H3K27me3	N/A	[46]
PU.1	Mouse	myeloid and lymphoid cells	myeloid and lymphoid development	MNase-seq and ChIP-seq	Create the macrophage-specific repertoire of accessible cis-regulatory elements	N/A	Promote H3K4me deposition.	N/A	[47,48]
p53	Human	Many	Tumor suppressor	EMSAs, ChIP-seq and ATAC-seq	Targets heterochromatin and binds to nucleosome in vitro	N/A	Promote H3K27ac and H4K16ac deposition	N/A	[49,50,51,52]
AP-1	Mouse	Many	Cell differentiation, proliferation and apoptosis	ChIP-seq and DNase-seq	Potentiate chromatin accessibility	N/A	N/A	N/A	[53]
ZELDA	Drosophila	Germ cells	Reprogramming specified germ cell to pluripotent cells (Zygotic genome activation)	FAIRE-seq	Open chromatin	N/A	N/A	Chromatin remains open even in the absence of ZELDA	[54,55]
GAF	Drosophila	Embryonic cells	Zygotic genome activation	ChIP-seq	Establish open chromatin and activate regulatory regions	N/A	Promote H3K4me1 deposition and H3K27me3 depletion	N/A	[56]
GRAINY	Human and Drosophila	Epithelial tissue	Epithelial cell-fate specification	ATAC-seq and ChIPmentation	Establish tissue-specific accessible chromatin landscapes	N/A	N/A	GRAINY binding open epithelial enhancers but not for gene activation	[57]
MYOD1	Mouse	Embryonic stem cells	Embryonic development	ATAC-seq, MNase-seq and ChIP-seq	Bind to inaccessible chromatin and open chromatin	N/A	Promote H3K37ac deposition	N/A	[39]
ESRRB	Mouse	Epiblast stem cells (EpiSCs)	Reprograming of EpiSCs to ESCs	ChIP-seq	Binds to silenced enhancers containing stable nucleosomes and hypermethylated DNA	Cooperative binding with OCT4, SOX2 and NANOG	Promote loss of DNA methylation and engagement of p300	N/A	[58]

**Table 2:** Pioneer factors in plants [26].

Pioneer TFs	Species	Organ/Cell Type	Biological Process	Identification	Pioneer Activity	Co-Factors	Epigenetic Effects	Other Features and Cautious Notes	References
LEAFY	*Arabidopsis thaliana*	Inflorescence meristem	Flower meristem establishment	ChIP-seq and RNA-seq	Bind to cognate sites in closed chromatin region	BRAHMA and SPLAYD	Counteract with PRC2 for H3K27me3 elimination	Oligomerization activity likely involve in targeting binding sites in closed chromatin	[59]
AP1	*Arabidopsis thaliana*	Flower organs	Flower organ specification	DNase-seq	Open closed chromatin				[60]
SEP3	*Arabidopsis thaliana*	Flower organs	Flower organ specification	DNase-seq	Open closed chromatin				[60]
LEC1	*Arabidopsis thaliana*	Embryonic cells	Vernalization	ChIP-qPCR	Establish stable epigenetic markers	N/A	Promote H3K36me3 deposition, and counteract with PRC2 for H3K27me3 elimination	N/A	[61]
